# Development of a patient reported outcome measures for measuring the impact of visual impairment following stroke

**DOI:** 10.1186/s12913-019-4157-3

**Published:** 2019-05-31

**Authors:** Lauren R. Hepworth, Fiona J. Rowe, Girvan Burnside

**Affiliations:** 1 Department of Health Services Research, University of Liverpool, Waterhouse Building Block B, 1-5 Brownlow Street, Liverpool, L69 3GL Liverpool, UK; 2 Department of Biostatistics, University of Liverpool, Waterhouse Building Block F, 1-5 Brownlow Street, Liverpool, L69 3GL UK

**Keywords:** Stroke, Brain injury, Visual impairment, Patient reported outcome measure, Development, Rasch analysis

## Abstract

**Background:**

Among the available patient-reported outcome measures (PROMs) there is an absence of a PROM with a specific focus on the impact of the wide variety of visual impairments following stroke.

Our aim was to develop a patient reported quality of life outcome measure for stroke survivors with visual impairment.

**Methods:**

Potential items were sourced from a combination of existing PROMs from a systematic review and qualitative in-depth interviews, duplicates were removed and items shortlisted. The initial pilot instrument was created following a ranking exercise of these potential items and consultation with stroke survivors. Version 1 was piloted with 37 stroke survivors at acute and chronic stages. Version 2 was piloted with 243 stroke survivors with visual impairment at acute and chronic stages. This data was analysed using the Rasch measurement model. Simultaneously, items from Version 2 underwent a Delphi process with stroke survivors and stroke clinicians, to assess the importance of each item. Final consensus decisions on item removal were made using the combined analysis from the Rasch measurement model and Delphi process in a nominal group meeting.

**Results:**

Due to the wide range of rank given to the majority of categories/items, only two items were discarded. Version 1 comprised of 102 items with 5 response categories relating to amount of difficulty. The pilot of Version 1 allowed item reduction based on analysis of floor/ceiling effects and not applicable responses. Version 2 comprised of 62 items. Within the nominal group meeting, the expert panel created a set of rules which aided them with decision making in addition to the Rasch and Delphi analysis data. This resulted in the removal of 43 items and the combination of seven items to create three new items. The expert panel also recommended the rewording of three items.

**Conclusion:**

The Brain Injury associated Visual Impairment Impact Questionnaire (BIVI-IQ-15), a 15-item instrument with 4 response categories has been developed for capturing vision-related quality of life of stroke survivors with any of the predominant types of visual impairment, in the presence of other impairments and for both inpatients and outpatients.

**Electronic supplementary material:**

The online version of this article (10.1186/s12913-019-4157-3) contains supplementary material, which is available to authorized users.

## Background

The prevalence of visual impairment following stroke has been reported as 72% [1]. Visual impairment as a result of stroke takes different forms across four main categories: visual field loss, ocular motility defects, reduced central vision and visual perception problems [2]. These impairments have the potential to affect an individual’s ability to perform activities of daily living (ADLs) for example self-care, mobility and socialising [3]. An individual with visual impairment may have reduced level of independence [4]. A combination of limitations has the potential to impact on an individual’s mood and motivation [5, 6].

A patient reported outcome measure (PROM) is “any report of the status of a patient’s health condition that comes directly from the patient, without interpretation of the patient’s response by a clinician or anyone else” [7]. How an individual feels and functions in relation to either their general health or a specific condition can be captured using PROMs. By allowing an individual to self-report using PROMs provides the opportunity for concepts to be captured, which with other methods is not possible [8]. PROMs can be used for a wide variety of purposes and can provide a vehicle for the patient’s voice, to inform clinicians and/or researchers with their views on the impact of their health status [9, 10].

Within clinical practice, stable non-recovered stroke-related visual impairment show no change on objective testing [2]. However, it is common for the stroke survivor to adapt to the visual impairment; the best way to capture this adaptation is by repeated measures of quality of life [3]. The availability of an instrument for measuring impact on quality of life for visual impairment following stroke would allow adaptation to be tracked formally from visit to visit. For example, a pilot randomised controlled trial of interventions for stroke-related visual field loss suggested vision-related PROMs assessing quality of life would be an appropriate primary outcome measure for this population [11]. Had quality of life not been measured in this trial, it would not have been highlighted that one of the treatments has the potential to improve quality of life in stroke survivors with homonymous hemianopia.

The need to develop a PROM specific to measuring the impact of the wide variety of visual impairment post-stroke was confirmed by the critic of the existing instruments available for measuring vision-related quality of life in a systematic review [12]. A further systematic review investigating the impact of stroke-related visual impairment found a wide variety of instruments being used in research [13]. For the studies in this review, it was not always possible to isolate the impact of the stroke-related visual impairment due to question wording. Other existing instruments pose a considerable burden on the person completing, due to the high number of items. This highlights the need for an appropriate robust instrument to measure vision-related quality of life for stroke survivors with visual impairment.

Our aim was to develop a patient reported outcome measure for stroke survivors with visual impairment to measure vision-related quality of life.

## Methods

### Development process

**Fig. 1:**
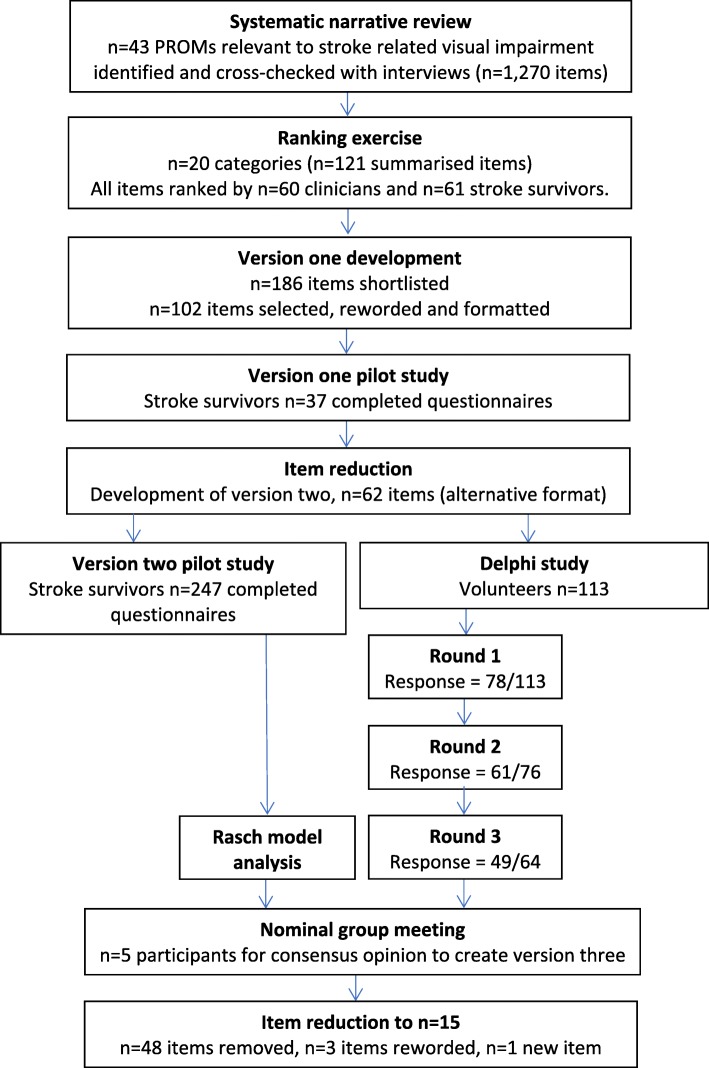
Flow chart of the instrument development process

The development process is outlined in Fig. 1. Ethical permission was granted by the West of Scotland Research Ethics Committee (14/WS/0090) and the University of Liverpool REC (IPHS-14145-040).

### Item generation

Items were sourced from a database created from the instruments found to be potentially relevant to stroke survivors with visual impairment in a systematic review of existing PROMs [12]. The total item pool was constructed of 1270 items. These items were coded, resulting in 20 categories. Lists of items were created by summarising the focus of the items within each category with the removal of duplications. This resulted in the following number of items in each category: walking (*n* = 9), near vision (*n* = 7), distance vision (*n* = 5), reading (*n* = 5), driving (*n* = 8), travelling (*n* = 4), television (*n* = 5), peripheral vision (*n* = 4), self-care (*n* = 8), lighting (*n* = 5), general vision (*n* = 6), well-being (*n* = 11), colour (*n* = 3), ocular pain (*n* = 3), social function (*n* = 9), role limitations (*n* = 8), dependency (*n* = 4), binocular vision (*n* = 5), symptoms (*n* = 11) and general health (*n* = 1).

The list of items was cross-checked against key themes and codes obtained from one-to-one interviews conducted with stroke survivors capturing their experience of their post-stroke visual impairment [14]. No additional items were identified.

### Ranking exercise

A ranking exercise was conducted to identify key items versus items not considered important for a new instrument. The stakeholders identified as being required included stroke survivors with visual impairment and eye specialists involved in stroke care (i.e. orthoptists). These groups were recruited by convenience sampling using voluntary sector (e.g. charities) advertisement and clinical meetings, respectively. Participants were requested to rank the categories and items in their perceived order from most important to least important; items which they felt were missing could be added. Clinicians completed the exercise on paper and stroke survivors completed a two-part web-based version.

### Version 1 development

In view of the results of the ranking exercise, the decision was made to include all 20 categories and a maximum of nine items per category. The limit in the number of items per category was to reduce the larger categories. This was not based on a standardised cut-off point across all categories; the variability in the number of items in each category prevented direct comparisons and, therefore, a standardised cut-off point. This resulted in the loss of one item from two categories; well-being and symptoms. ‘Adaptation’ was removed from the well-being category and ‘starbursts’ from the symptoms category. Stroke survivors and clinicians were in agreement with the low ranking of these items.

Following the removal of duplicates, items were shortlisted by the research team if found to be appropriate; 1) in terms of language  (e.g."driving...during rush hour on the freeway" whereas motorway would be the English terminology), 2) not being too detailed on the specifics of an activity (e.g. "meal preparation - chop, slice, cut, peel, use knives safely") and 3) not being too specific on the location of an activity (e.g. “moving about in classrooms”). The short-list included multiple items under several an item headings used in the ranking exercise; for example, ‘watching TV’ had six short listed items. Version 1 was constructed of 102 items, outlined in Table 1, selected from the shortlisted items by the research team in consultation with stroke survivors. A decision was also taken by this group as to how the items in the new instrument should be worded. This decision was based on recommendations from the analysis of existing instruments [12, 13]. All items asked about “difficulty due to eyes or eyesight” to support the purpose of the instrument. The scale was standardised, using a five-point rating scale measuring the level of difficulty [15]. The scale ranged from 1 ‘none at all’ to 5 ‘it limits my activity’, with an additional ‘not applicable’ option. A box was also provided to allow explanation of why an item was not applicable. The exception to this was for the two overall items, ‘general health’ and ‘rate eyesight’ - both items used the same wording and a visual analogue scale ranging from zero (worst possible) to 100 (best possible).

**Table 1:** Items included in Version 1

i. General health	33. Poor light	67. Strangers
ii. General vision	34.Bright light	68. Social activites
1. Blurred vision	35. Dim to bright	69. Entertaining
2. Distortion	36. Bright to dim	70. Outdoor activities
3. Objects jumping	37. Haloes	71. New friends
4. Deterioration	38. Recognising colours	72. Usual activities
5. Fluctuation	39. Clothes	73. Confidence
6. Tiredness	40. Dull colours	74. Accomplishing
7. Two eyes different	41. Pain	75. Limiting how long
8. Double vision	42. Strained	76. Limiting opportunities
9. Judging distance	43. Headaches	77. Usual standard
10. Unusual appearance	44. Dryness	78. Toilet
11. Recognising people	45. Watering	79. Dressing
12. Reading signs	46. Steps	80. Eating
13. Reading bus numbers	47. Tripping	81. Medication
14. Clock	48. Crossing road	82. Pouring drink
15. Recognising faces	49. Familiar areas	83. Preparing food
16. Writing	50. Unfamiliar areas	84. Looking after appearance
17. Work/hobbies	51. Crowded areas	85. Household chores
18. Finding	52. Indoors	86. Shopping
19. Money	53. Outdoors	87. Bathing
20. Watch	54. Uneven	88. Sad
21. Telephone	55. Ever driven	89. Frustrated
22. Finding next line	56. Driving during daytime	90. Vulnerable
23. Ordinary size	57. Driving at night	91. Anxious
24. Small print	58. Seeing cars in next lane	92. Worry
25. Large print	59. Driving in difficult conditions	93. Isolated
26. Watching TV	60. Oncoming headlights	94. Less control
27. Reading text	61. Parking	95. Stressed
28. Cinema	62. Car passenger	96. Not coping
29. Computer	63. Alone	97. Self-conscious
30. Suddenly appearing	64. Public transport	98. Burden
31. Missing patches	65. Meeting family/friends	99. Help from others
32. Objects to side	66. Eye contact	100. Stay at home

### Version 1 pilot

Version 1 was prospectively piloted with 1) acute stroke survivors recruited at three NHS hospitals during routine clinical appointments for visual problems and 2) long-term stroke survivors recruited through voluntary sector channels. Participants were included if over 18 years of age and had a clinical or radiological confirmed stroke with related visual impairment, and excluded if they were unable to give informed consent. Both participant groups were asked to complete version one of the new instrument and a feedback form. The feedback form consisted of six questions about the clarity of instructions, repetition of questions, the response scale aimed to collect the views of the participants on completing the questionnaire. The questionnaire and feedback form were completed in paper format. If required by the participant the questionnaire was delivered in interview format by the clinician.

### Version 1 item reduction & version 2 development

Analysis on the data collected as part of the version 1 pilot was conducted, focusing on the spread of responses, to identify items with large floor and ceiling effects, not applicable responses and inter-item correlation. Only a high inter-item correlation (> 0.8) would be highlighted to the nominal group at this stage. Items with having high inter-item correlation is suggestive that the items are in effect duplications [16].

### Version 1 to version 2 amendments

The data collected in the pilot of version 1 was used in a nominal group session to develop version 2. Each section and item were discussed individually in terms of response frequencies, inter-item correlations, item wording and participant feedback. The process of the meeting followed three steps:

1. idea sharing,
2. group discussion and clarification,
3. decision agreement.

### Version 2 pilot

Version 2 was piloted with acute and long-term stroke survivors recruited from 11 NHS hospitals and voluntary sector organisations in the same manner as for version 1. Participants were included if over 18 years of age and had a clinical or radiological confirmed stroke with related visual impairment, and excluded if they were unable to give informed consent. Participants who had completed version 1 were not approached to complete version 2. The questionnaire was completed in paper format. If required by the participant the questionnaire was administered in interview format by the clinician.

### Delphi process

Alongside the clinical pilot of version two, the 62 items were also used to create a reactive three-round electronic Delphi survey. This asked clinicians and stroke survivors to rank each item in terms of importance on a 9-point scale from 1 ‘not important’ to 9 ‘critical’ and categorise the items by relevance to types of visual impairment following stroke or not relevant. Analysis of consensus, stability, and agreement was conducted. The detailed methods of this component of the development process are published elsewhere [17].

### Rasch analysis

Rasch analysis aims to maximise homogeneity of the trait being measured and redundancy reduction with as little impact on the measurement information by removal of items and/or amendment of scoring levels to create the most valid and simple measure possible [18].

The Rasch measurement model was used to evaluate the 62-item version 2. Analyses were performed using RUMM 2030 software (RUMM Lab, Australia) [19]. The unrestricted ‘partial credit’ Rasch polytomous model was used [20]. Items were removed from the scale and the resultant scale was reassessed for fit, differential item functioning (DIF), dimensionality and local dependency. Item removal was an iterative process which required repetition of these stages until an adequate solution for the scale was found.

The following person factors were included along with individual item responses for DIF analyses: age (< 65 or > 65 years), gender (male or female), visual impairment diagnosis (reduced central vision, ocular motility defect, visual field defect or visual perception problem), number of visual impairments (isolated or multiple), location (inpatient or outpatient) and time since stroke (hyper-acute, acute, sub-acute or long-term). This enabled analysis of the instrument to establish if the items worked in the same way irrespective of these factors. It was deemed important that the instrument be suitable and without bias for: those of working age and retired, the variety post-stroke visual impairment and stroke survivors at different stages post-stroke.

**Fig. 2:**
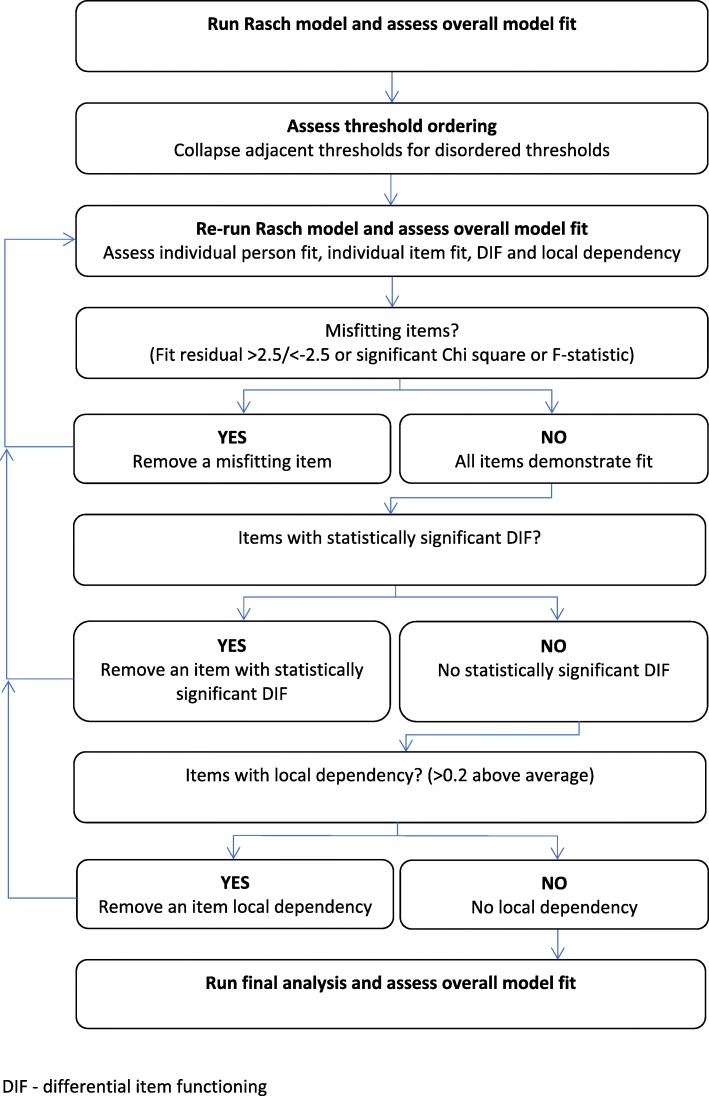
Methodological stages of Rasch Model analysis, all stages completed as part of the initial process prior to any changes being made.

A flow chart of the Rasch analysis process is outlined in Fig. 2. All stages of analyses were conducted as part of the initial process of testing the instrument prior to any changes being made. The following criteria were used in the analysis:

- Disordered thresholds indicate that the scoring categories are not working properly (participants are not responding as predicted) which could be a result of too many category options or the semantics of the category labels being confusing [21].
- The individual person fit was assessed to identify any individual persons that are misfitting, which could skew the analysis [22]. Individuals who responded in the expected way would fall within a commonly accepted fit residual range of − 2.5 to + 2.5 [23–25]. Misfitting persons were not removed during this analysis.
- Individual item fit was assessed formally using three statistics; fit residuals, chi-square probability and F-statistic. Items which are working as expected have commonly accepted fit residuals within the range of − 2.5 to + 2.5, a non-significant (*p* > 0.05) Chi-square and F-statistic [23, 24, 26].
- The presence of DIF relating to the person factors included is indicated with a significant result (*p* < 0.05).
- Local independence is violated when responses to items are interrelated to each other [27]. Dependency between items changes the probabilistic structure and can cause an overestimation of construct validity and reliability of the instrument [24, 28]. Local dependence can be identified using residual correlations of the items. A cut-off point of 0.2 above the average of all item residual correlations was used to identify local dependence in these analyses [24, 27].
- Unidimensionality is a principle of measurement in which only one attribute is measured at any one time. Principal Component Analysis (PCA) is conducted using fit residuals (the differences between the expected and observed responses) for each person and each item [29]. Person estimates (ability) are generated from two subsets of items (most negative loading residuals and most positive loading residuals) and compared against each other on an individual person basis using paired t-tests [29, 30]. If > 5% of t-tests run are statistically significant (< 0.05), multidimensionality is indicated [29].

There are numerous reasons to consider the removal of an item; poor item fit, DIF, local dependence and multidimensionality. In view of the aim of the new instrument to be appropriate for all stroke-related visual impairment at all stages post-stroke, during this analysis the method of dealing with DIF was item deletion. Removing items changes the relationship between the remaining items and the model. Therefore this process was conducted in an iterative manner with the removal of only one item at a time [30, 31]. Following the removal of any item the analysis was rerun, as outlined in Fig. 2.

### Final consensus process

To finalise the item removal decisions with input from stroke survivors and clinicians, both the three-round Delphi survey and Rasch analysis were presented at a nominal group meeting. The session was organised into ten tasks.

Each participant was given the data sets relevant for tasks one to eight. The data sets included response histograms from the final round of the Delphi survey, and explanations relating to Rasch analysis findings.

The following steps were used for each task [32]:

1. Verbal and written presentation of the data set with questions to consider.
2. Generation of ideas and opinions in silence.
3. Feedback from each participant in turn to the group, listed by the facilitator on a flip-chart for reference during the discussion.
4. Group discussion regarding the feedback.
5. Voting and decision agreement regarding inclusion or exclusion of items.

The consensus definition used for the session when voting was an acceptable resolution, one that can be supported, even if not the ‘favourite’ of each individual.

Tasks one to three dealt with items which required exclusion during Rasch analysis due to either individual item misfit or differential item functioning (DIF). The participants were asked to consider three questions for each of the items within these first three sets:

- Are these items important to measuring vision-related quality of life?
- If an item is important, is the topic covered by another item?
- Can this item be excluded?

Tasks four to eight dealt with items for which local dependence had been detected. The participants were given the items in groups which had been found to have local dependence and asked to choose which item(s) could be excluded.

## Results

### Ranking exercise

Fifty-nine orthoptists, one ophthalmologist and 61 stroke survivors participated in the ranking exercise. The web-based version completed by stroke survivors was divided into two parts which caused some difficulties with completion. This resulted in 21 completions for the whole ranking exercise. Fifty-nine responded to part one of which, 18 were incomplete, and 25 responded to part two, of which four were incomplete. All complete and incomplete responses were used to maximise the available information [33].

Both the category and individual item rankings displayed a wide range of ranks. Stroke survivors gave the full range of rank for all items within a category, for nine out of twenty categories. Clinicians used the full range of rank for all items within a category for eight out of twenty categories. Only in four of the twenty categories did both stroke survivors and clinicians use the full range of rank for less than half the items [33].

### Version 1 pilot

Following 12 months of recruitment (July 2014 to June 2015) 37 questionnaires were returned from 52 recruited participants (71.2% return rate), from. A decision was taken that the lack of recruitment was most likely due to the high number of items (*n* = 102) within the instrument. Due to the low number of returns, Rasch analysis was not possible at this stage.

The participant feedback received regarding version 1 supported the need for item reduction. Of those who completed version one of the pilot instrument, 15 participants (40.5%) returned a feedback form. The majority (73.3%, *n* = 11) reported that the instructions were clear. There was an equally split view on whether the instrument had repetitive items. The majority (66.7%, *n* = 8) reported that no change was required to the scale. Of those that reported the scale should be changed the comments included for example, “a little bit and moderate are hard to define”.

In view of both the low recruitment rate and participant feedback, a first round item reduction was required to increase subsequent recruitment.

### Version 1 item reduction & version 2 development

#### Floor-ceiling effects

Floor effects were apparent within the colour category (3 items) with the percentage response as option 1 ‘none at all’ ranging from 62.2 to 75.7%. Within the self-care category, eight of the ten items (excluding ‘household chores’ and ‘shopping’) had floor effects ranging from 62.2 to 78.4%. The largest ceiling effect was found in the ‘ever driven’ item; 54.1% responded choosing the maximum score equivalent to ‘so much I can’t do this activity’. The only other option chosen for this item was equivalent to ‘not applicable’. The remaining items used the full range of responses.

### Not applicable option

Twenty-four of the items had not applicable response rates which would exceed an acceptable level of missing data of < 10% [34].

Five items had a 13.5% not applicable response rate; ‘crossing the road’, ‘walking on uneven ground’, ‘social activities’, ‘entertaining at home’ and ‘making new friends’. A further six items had a 16.2% not applicable response rate; ‘doing work and hobbies’, ‘telling time on a wristwatch’, ‘noticing haloes’, ‘walking outdoors’, ‘limiting opportunities’ and ‘working to usual standards’. All items within the travelling category had high not applicable ratings ranging from 24.3 to 29.7%. A further five items had higher not applicable response rates; the highest being item ‘ever driven’ with 43.2%, followed by ‘watching a film at the cinema’ with 37.8%, ‘using a computer’ with 27.0%, ‘outdoor activities’ with 24.3% and ‘reading bus numbers’ with 18.9%.

In cases where an item was not applicable to the participant, they were asked to state the reason the item was not applicable. From both the acute and long-term stroke survivors a total of nine codes emerged; do not do this activity (*n* = 73), not tried this activity yet (*n* = 41), still an inpatient (*n* = 31), can do with help (*n* = 26), do not experience this problem (*n* = 23), problem caused by other difficulty (*n* = 18), not working/retired (*n* = 11), adaptation (*n* = 8) and did not understand the question (*n* = 8).

### Version 1 to version 2 amendments

One stroke survivor, one orthoptist and two statisticians attended the nominal group session plus the facilitator.

A total of 29 items were combined to form 10 new broader items, 16 items were reworded, and 21 items were removed. A summary of the changes agreed are outlined in Table 2.

**Table 2:** Summary of changes made to version one to create version two of the new instrument

Version one		Changes Made	Reason	Version two	
Section heading	No of items			Section heading	No of Items
General vision	10	• ‘Blurred vision’ and ‘distortion’ merged	• High correlation 0.801	General vision	9
Distance vision	4	• All items replaced by general distance vision items ‘difficulty seeing far side of a room’ and ‘difficulty seeing far away’	• 4 items in version 1 refer to specific tasks. ‘Bus numbers’ high N/A response	Distance vision	2
Near vision	7	• Wording change in ‘recognising faces and seeing facial expressions’ to ‘seeing faces and facial expressions’	• Original wording related to prosopagnosia	Near vision	5
		• ‘Doing work or hobbies’, ‘identifying coins and bank notes’, ‘telling time on a watch’ and ‘using a telephone’ replaced by new item ‘difficulty with close up vision’.	• 4 items in version 1 refer to specific tasks		
		• Addition of ‘difficulty using a computer’ item from television section	• Item relates to near vision		
Reading	4	• ‘ordinary print’ ‘small print’ and ‘large print’ items replaced by ‘reading same size print as before’	• New item isolates impact of new stroke related visual impairment	Reading	2
Television	4	• ‘Watching television’ and ‘reading text on television’ removed used as example for ‘difficulty seeing far side of a room’ item in distance vision section	• Items related to distance vision activities	–	Section removed
		• ‘Watching a film at the cinema’ removed	• High N/A response 26.9%		
		• ‘Difficulty using a computer’ item reworded and moved to near vision section	• Item relates to near vision		
Peripheral vision	3	No changes made	–	Peripheral vision	3
Lighting	5	• ‘Adjusting to brightness from dim light’ and ‘adjusting to darkness from bright light’ combined to create ‘adjusting to differing lighting’	• High correlation 0.810	Light	4
		• ‘Haloes’ item removed	• Floor effect 44.2% reporting no issue and 11.5% N/A response		
		• Addition of ‘change in colour’ from colour section			
Colour	3	• All items replaced by ‘change in colour’ item	• All items floor effects (‘recognising colour’ 70.2%, ‘picking out clothes’ 75.6%, ‘colours appear dull’ 62.1%)	–	Section removed
		• ‘Change in colour’ item moved to light section	• New item isolates impact of new stroke related visual impairment		
Discomfort	5	• ‘Pain and discomfort’, ‘headaches’ and ‘eyes feeling strained’ items removed	• Difficult to attribute cause and therefore to answer accurately	Discomfort	2
Walking	9	• ‘Steps, curbs and stairs’ item combined with ‘uneven ground’ item	• Similar situations	Moving around	9
		• ‘Tripping and falling’ and ‘bumping into’ items reworded	• Context added		
		• Walking replaced by moving around in all items	• Reworded to include mobilisation by other methods e.g. wheelchair		
		• Addition of ‘travelling as a passenger’ item from travelling section	• Now section not specific to walking		
Driving	7	• All items removed	• Floor and ceiling effects all participants had either never driven or given up driving	–	Section removed
Travelling	3	• ‘Travelling alone’ and ‘traveling on public transport’ items removed	• High correlations 0.786 and 0.761 with ‘travelling in a car as a passenger’	–	Section removed
		• ‘Travelling in a car as a passenger’ reworded to incorporate ‘travelling on public transport’ and moved to moving around section			
Socialising	7	• ‘Visiting family and friends’, ‘entertaining in your home’ and ‘making new friends’ items removed	• High correlations 0.773 and 0.776 identified duplication with ‘social activities’	Socialising	4
		• Wording of the ‘social activities’ and the ‘outdoor activities’ items combined to create two items, ‘indoor social activities’ and ‘outdoor social activities’.	• Differentiation between indoor and outdoor activities		
Role limitations	6	• ‘Performing usual activities’ and ‘people limiting your opportunities’ items removed	• High correlation 0.816 with ‘doing usual work to usual standard’ and N/A response 11.5%	Role limitations	4
		• ‘Doing usual work to usual standard’ and ‘limit of how long you can work’ reworded			
			• N/A response of not working/retired 11.5%		
Self-care	10	No changes made	–	Independent living	10
Well-being	13	• ‘Feeling sad and low’, ‘frustrated’, ‘anxious’, ‘worry’, ‘feeling isolated’, ‘feeling less control’ and ‘stressed’ combined into one item ‘negative emotions’	• High correlations 0.810 to 0.929	Well-being	6
			• High correlation 0.810		
		• ‘Feeling a burden’ and ‘needing help from others’ combined into one item, ‘feeling a burden’			

The outcome of the nominal group meeting was the production of version 2; constructed of 62 items.

To improve the usability of the PROM, the formatting of version 2 was altered following comment in the version 1 feedback form and the stroke survivor delegate at the nominal group meeting. Alterations reduced the repetition of “due to your eyes and eyesight” to once per page rather than for every question.

### Version 2 pilot

A total of 275 participants were recruited over a 17-month period; 236 participants from NHS hospitals and 39 participants through the voluntary sector. A total of 247 questionnaires were returned (89.8% return rate).

Of the 247 stroke survivors recruited who returned a completed questionnaire 59.9% (*n* = 148) were male and the mean age at time of recruitment was 68.8 years (SD 13.0). The median number of days since stroke onset at time of recruitment was 45 days (IQR 10–207) and the mean 493.0 days (SD 1474.7); the discrepancy due to a number of individuals recruited from outpatient clinics several months or years post-stroke. The mean Barthel Index score as an indication of stroke severity for those recruited by NHS hospitals was 14.4 (SD 5.9), indicating a moderate dependency on average [35, 36]; 47.9% (*n* = 101) were inpatients when recruited and 52.1% (*n* = 110) were outpatients. Sixty-eight percent (*n* = 143) of the stroke survivors recruited by NHS hospitals who returned a questionnaire had two or more visual impairments. The types of visual impairment of those that returned a questionnaire are outlined in Table 3. The most commonly reported symptom was visual field loss (52.6%, *n* = 111), followed by reading difficulties (31.8%, *n* = 67).

**Table 3:** Types of visual impairment in acute stroke survivors returning a questionnaire

Visual impairment	*n* (%)
Visual field loss	182 (86.3)
Ocular motility defect	104 (49.3)
Central vision loss	77 (36.5)
Ocular alignment defect	44 (20.9)
Visual inattention	26 (12.3)
Visual perception problems	5 (2.4)

### Delphi process

One hundred and thirteen participants registered for the Delphi survey; 47 (41.6%) completed all three rounds. Consensus was reached on importance of 56.5% of items in the three-round process; all as ‘critical’ (options 7–9) - an indication for inclusion in the instrument. A consensus was reached for 83.8% in the categorisation of items. The majority (82.6%) of consensus were for relevant to ‘all visual impairment following stroke’. However, two items were deemed ‘not relevant’ and therefore potentially suitable for removal. The detailed results of this component of the development process are published elsewhere [17].

The lack of item reduction achieved by the Delphi process alone highlighted the need for additional methods of item reduction.

### Rasch analysis

The initial model fit for the scale was poor (χ^2^ = 2110.3, *p* < 0.0001). The initial analysis demonstrated 46 misfitting items, 156 instances of local dependency, 17 items with DIF and 61 items with disordered thresholds. Prior to any further analysis, the thresholds were reordered. Reordering was achieved by combining response categories together, using the category probability curves, category response frequencies and the nomenclature of the categories as guides to which categories should be combined whilst maintaining as many thresholds as possible [37].

No items retained the original threshold order. The two items with visual analogue scales were reduced from 101 options to 11 (ten thresholds), as the majority of participants (89.9%) had selected a round number. Of the 60 items which originally had a 5-point rating scale (four thresholds), ten items reduced the number of thresholds to three, 47 items reduced to two and three items reduced to dichotomous (one threshold).

Following threshold ordering the model fit improved but remained poor (χ^2^ = 753.10, *p* < 0.0001), now demonstrating 11 misfitting items, 102 instances of local dependency, 12 items with DIF and 36.0% significant paired t-tests indicating multidimensionality. Item fit analysis showed eight had fit residuals either greater than 2.5 or less than − 2.5, six had a significant Chi-square result and ten had a significant F-statistic.

The order of item deletion is listed in Additional file 1, along with the summary statistics of the analysis following each item deletion. Item deletion began with the individual items identified as misfitting, with the order of deletion was led by the degree of misfit. The order and the reason for deletion is outlined in Additional file 2.

Following deletion of 43 items the summary statistics improved to indicate the instrument had achieved fit with the Rasch model (Additional file 1). The fit residual means were close to zero and the standard deviation close to one for item fit (mean − 0.193, SD 0.966) and person fit (mean − 0.275, SD 1.235). The Chi-square item-trait interaction statistic result was non-significant with Bonferroni correction (*p* = 0.0332). Unidimensional was indicated by 3.24% significant paired t-tests (< 0.05). The person separation index of the current 19-item instrument was 0.84 (Cronbach’s alpha 0.90) exhibiting good interval validity.

**Fig. 3:**
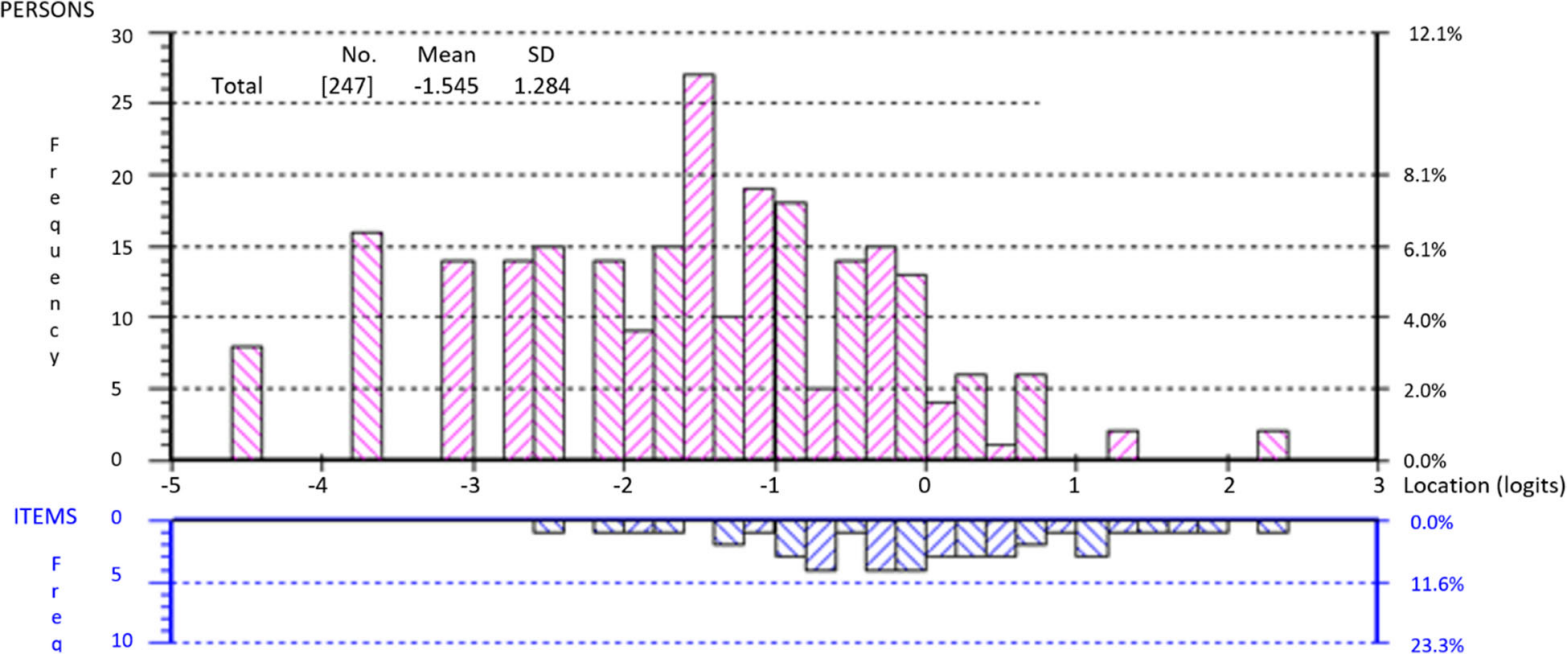
Person-item threshold distribution of the 19-item instrument following item deletion achieving fit to the Rasch model and unidimensionality; a graphical representation of targeting

Targeting for the 19-item instrument is represented in Fig. 3. The negative logit value of the person location (mean − 1.545, SD 1.284) indicates the sample population have experienced a higher vision-related quality of life than the average of the scale, presenting evidence of a ceiling effect. Despite this apparent mistargeting, less than 5% of the sample population had extreme scores.

The analysis revealed a mistargeting of the instrument, suggesting insufficient items to differentiate between participants with a better vision-related quality of life. This could be the result of participants with asymptomatic mild visual impairment (e.g. partial superior homonymous quadrantanopia) or a visual impairment for which the symptoms had been eliminated by treatment (e.g. diplopia joined with a prism).

During the process of Rasch analysis the psychometrics led the majority of decisions on which items to remove and in which order. However, on occasion, clinical judgements were made by the research team when deciding which items to delete in cases of local dependence, resulting in some item selection being subjective. The reasons for these decisions are outlined in the clinical column of Additional file 2. Rasch analysis selects the items with the best statistics, but this does not necessarily translate to the best items to clinically measure vision-related quality of life of stroke survivors [38].

### Final consensus process

Fourteen of the 47 participants who completed the third round of the Delphi survey expressed an interest in participating in the nominal group meeting (two stroke survivors, six orthoptists and six occupational therapists (OT)). Five participants were able to attend the meeting (two stroke survivors, two orthoptists and one OT) to form the expert panel plus the facilitator. The group followed the ten tasks outlined in Table 4.

**Table 4:** Overview of tasks for the nominal group process

**Task One** – Misfitting items	
‘Deterioration’	‘Dealing with strangers’
‘Eyes seeing differently’	‘Participating in indoor social activities’
‘Dry eyes’	‘Loss of confidence’
‘Watery eyes’	‘Limit of how long activities can be done for’
‘Making eye contact’	‘Household chores’
**Task Two** – Items with DIF for location and time since stroke person factors	
‘Overall health’	‘Crossing the road’
‘Overall vision’	‘Moving around in unfamiliar areas’
‘Using a computer’	‘Bumps into or against objects or people in
‘Seeing in bright light’	crowded areas’
‘Moving around on uneven ground’	‘Moving around outdoors’
	‘Stay at home’
**Task Three** – Items with DIF for primary visual impairment person factor	
‘Double vision’	‘Missing patches of vision’
‘Objects suddenly appearing’	‘Noticing objects off to the side’
**Task Four **– Items with local dependency in the general and distance vision categories	
‘Blurred vision’ OR ‘Objects jumping around’ OR ‘Fluctuation’	
‘Blurred vision’ OR ‘Seeing something far away’	
‘Seeing far side of a room’ OR ‘Seeing something far away’ OR ‘Seeing faces’ (Delete 2)	
**Task Five** – Items with local dependency in the near vision and reading categories	
‘Following a line of print’ OR ‘Reading same print size’	
‘Writing’ OR ‘Close-up vision’ OR ‘Following a line of print’	
**Task Six** – Items with local dependency in the lighting, moving around and role limitation categories	
‘Seeing in poor or dim lighting’ OR ‘Adjusting to differing lighting’ OR ‘Change in colour perception’ (Delete 2)	
‘Accomplishing as much as you would like’ OR ‘Usual standard’	
‘Moving around in familiar areas’ OR ‘Moving around indoors’	
**Task Seven** – Items with local dependency in the independent living and moving around categories	
‘Toileting’ OR ‘Getting dressed’ OR ‘Preparing something to eat’ OR ‘Bathing or showering’	
‘Getting dressed’ OR ‘Preparing something to eat’ OR ‘Taking medication’ OR ‘Looking after your appearance’ OR ‘Pouring a drink’ OR ‘Shopping’ (Delete 3)	
‘Eating’ OR ‘Pouring a drink’	
**Task Eight** – Items with local dependency in the well-being category	
‘Negative emotions’ OR ‘Vulnerable’ OR ‘Burden to others’	
‘Not coping’ OR ‘Self-conscious’	
**Task Nine** – Overview of remaining items	
All remaining items - any further exclusions required?	
‘Objects jumping around’	‘Travelling as a passenger’
‘Tired eyes’	‘Making eye contact’
‘Judging distances’	‘Participating in indoor social activities’
‘Unusual appearance’	‘Participating in outdoor social activities’
‘Seeing something far away’	‘Loss of confidence’
‘Close-up vision’	‘Usual standard’
‘Finding something’	‘Getting dressed’
‘Using a computer’	‘Looking after your appearance’
‘Following a line of print’	‘Feeling negative emotions’
‘Adjusting to differing lighting’	‘Not coping’
‘Tripping and falling’	‘Feeling a burden’
‘Moving around indoors’	
**E-Nominal Group**	
‘Eating’ OR ‘Looking after appearance’	
‘Close up vision’ OR ‘Following a line of print’	

The group unanimously agreed to remove seven of the ten misfitting items. The reasons given for removal included the Delphi results suggesting items were not relevant to stroke-related visual impairment, items not being vision-specific, lack of clarity of the question and covered better elsewhere in the instrument. A decision could not be agreed on the remaining three (‘making eye contact’, ‘participating in indoor social activities’ and ‘loss of confidence’), therefore these were held for discussion in task nine. Thirteen of the 14 items identified with DIF had unanimous agreement for removal to allow comparison between inpatient and outpatient responses and across different types of visual impairment. The exception was ‘using a computer’; it was viewed as an important item to future proof the instrument. The removal of a further 16 items was agreed due to local dependency. The reasons given for removal included the level of importance from Delphi results, function based items were clearer than symptom based items, potential crossover with other stroke sequelae and the item not being vision-specific, duplication, covered better elsewhere in the instrument and ensuring all questions relevant for both inpatients and outpatients.

In task nine during the discussion of all remaining items, an additional five items were excluded: ‘objects jumping around’, ‘unusual appearance’, ‘travelling as a passenger’, ‘making eye contact’, ‘bathing and showering’. Seven items were combined to form three items: 1) ‘participating in indoor social activities’ and ‘participating in outdoor social activities’ created ‘socialising’, 2) ‘accomplishing as much as would like’ and ‘usual standard’ created ‘doing what you want to do’, and 3) ‘loss of confidence’, ‘not coping’ and ‘feeling a burden’ created ‘doing things for yourself’.

The instrument with agreed removal decisions was reanalysed again using the Rasch measurement model. The wording of six items was altered during the nominal group process. However, for the purpose of further analyses the origin of the new item was used. No items indicated misfit and the instrument was unidimensional. Furthermore, four items displayed two residual incidences of local dependence: ‘eating’ and ‘looking after appearance’, ‘close-up vision’ and ‘following a line of print’. These two options were put to the expert group, who agreed to remove ‘eating’ and ‘close-up vision’. Local independence was achieved with the removal of these two items.

Through the nominal group process, the expert panel created a set of rules which aided them with decision making. First, that the instrument should be applicable to both inpatient and outpatient populations. Another rule was to shape the instrument using items which were important when measuring vision-related quality of life; this included the exclusion of symptom-related items. The group considered that difficulty with symptoms did not necessarily correspond to an impact on quality of life. The group favoured items which focused on specific activities which individuals either need to perform, or do for enjoyment.

The instrument was reduced to 15 items, which are listed in Table 5. In addition to reducing the number of items within the instrument, the expert panel also refined the wording of five items. This was for a number of reasons: 1) to clarify what the question was asking, as was the case with the ‘trips and falls’ item, 2) to dispense with the specifics of indoors and outdoors, as with the socialising items, and 3) to enable a new item to be created from the combination of two or more items, which was the case in the creation of ‘doing things for yourself’.

**Table 5:** Items to be included in the final version of the new instrument

How much difficulty do you have, due to your eyes or eyesight with?	
1. Tired eyes	
2. Judging distances	
3. Seeing something far away	
4. Finding something	
5. Using a computer	
6. Following a line of print	
7. Adjusting to differing lighting	
8. Fear of tripping and falling	
9. Getting about	
10. Socialising	
11. Doing what you want to do	
12. Getting dressed	
13. Looking after your appearance	
14. Feeling negative emotion	
15. Doing things for yourself	

Rasch analysis was used to assess which best suited the model; a 3-point or a 4-point rating scale. For the purpose of this analysis the origin of the new item was used, as was done for the post-nominal group analysis. The model fit and unidimensionality were stronger using the 4-point rating scale (three thresholds) across all the items. The research team therefore decided to reduce the number of response options for all items from five in version two to four in version three. The resulting rating scale scores each item from 0 ‘none’ to 3 ‘stops what I can do’, with the no response scored as 0 (perceived as no difficulty experienced). The maximum possible score on the 15-item scale would be 45, indicating the highest impact.

## Discussion

The development of the new instrument employed consultation with stroke survivors and clinicians throughout the development process, including item identification, item selection and scoring, in order to create an instrument suitable for measuring the impact on quality of life caused by the visual impairments associated with stroke.

This paper has reported the process of developing a new measure to capture vision-related quality of life of stroke survivors with visual impairment. Although this instrument was developed with and for stroke survivors, there is the potential to broaden the target population of the new instrument to include brain injury and other neurological conditions. This is based upon the fact that the visual sequelae of stroke is broad and can include visual field loss, ocular motility defects, reduced central vision and visual perception problems and stroke can also result in numerous other sequelae occurring simultaneously, such as physical disability, communication problems and cognitive impairments [39–43]. The visual impairments associated with a stroke can also result from other neurological aetiologies, such as traumatic brain injury (TBI), space occupying lesions, vascular, inflammation, e.g. multiple sclerosis (MS), infection, e.g. meningitis and degeneration, e.g. Parkinson’s disease. These conditions can also be associated with other global signs and symptoms affecting mobility, communication and cognition. The 15-item instrument was therefore named the Brain Injury associated Visual Impairment Impact Questionnaire (BIVI-IQ-15).

A systematic review of the impact of visual impairment following stroke on quality of life identified sub-categories which displayed reduced scores in existing PROMs [13]. This allows a comparison of BIVI-IQ-15 to existing vision-specific instruments. The NEI VFQ-25 used by five studies had six common subscales with reduced scores for stroke survivors with visual field loss compared to healthy individuals: general health, general vision, near activities, vision-specific mental health, driving and peripheral vision [44–48]. Of these six subscales, the BIVI-IQ included items related to three. The sub-categories not included (general health, driving and peripheral vision) had all been included in either version one or two of the instrument, and subsequently removed for a variety of reasons; i.e. not relevant to the majority of the target population or not relevant to measuring vision-related quality of life.

One study included in the review had a study population with reduced visual acuity in addition to visual field loss. As a consequence, the list of sub-categories with reduced scores was extended: general vision, near vision, distance vision, social functioning, vision-specific mental health, role difficulties and dependency [44]. The BIVI-IQ contains items covering all these sub-categories. This demonstrates that version three has incorporated items relevant to the sub-categories which potentially reveal the impact of visual impairment following stroke.

It must be highlighted that the examples of these studies used as comparison were predominantly stroke survivors with visual field loss rather than the broader spectrum of post-stroke visual impairment. There were no studies found in the review which investigated the impact of ocular motility defects using a vision-specific PROM. One study has used the NEI VFQ-25 to assess vision-related quality of life in a small sample with spinocerebellar ataxia of which 63.2% of participants had at least one ocular motor defect [49]. They found reduced scores when compared to a normal reference population in the following sub-categories: general vision, near vision, distance vision, driving, peripheral vision, vision-specific role difficulties, dependency, social functioning and mental health. These subcategories are similar to those raised with co-existing visual field loss and reduced central vision [44]. The BIVI-IQ has items which would be grouped into seven of these nine sub-categories. The two exceptions are driving and peripheral vision; the reasons for these disparities are explained above. This comparison has exhibited that the BIVI-IQ has face validity, as it contains items in sub-categories which have previously demonstrated an impact of visual impairment associated with a neurological cause.

In terms of size and the number of items, the BIVI-IQ is the smallest of the vision-related PROMs previously used with stroke survivors. Using the quality assessment modified from Pesudovs et al. and Hamzah et al., which was completed on all relevant existing PROMs as part of the systematic review of existing instruments [50, 51], the BIVI-IQ scored 13 out of 14 as demonstrated in Table 6. This is equivalent to the Activity Inventory (AI), the Daily Tasks Dependent on Vision (DLTV) questionnaire and the Veterans Affairs Low Vision Visual Functioning (VA LV VFQ) questionnaire [12]. In this systematic review, both the AI and DLTV were found to have a serious flaw in the question wording when using the instrument with individuals having co-existing non-ocular deficits, in that there was no reference to vision or eyesight [52, 53]. The VA LV VFQ had a potential high task burden with up to 192 items.

**Table 6:** Quality assessment of BIVI-IQ using the modified quality assessment tool for evaluation of PROMs from Hepworth et al. (2015) [12]

Quality criteria	Definition	BIVI-IQ
Pre-study hypothesis	The pre-study specification of the aim of the instrument and the intended population	**√√** A clear description is provided of the aim of the instrument and the intended population
Intended population	The extent to which the instrument has been studied in the intended population	**√√** Intended population studied
Actual content area	The extent to which the content meets the pre-study hypothesis specifications	**√√** Content is intended and is relevant to the intended population
Item identification	Selection of the items relevant to the target population for inclusion in the pilot instrument	**√√** Comprehensive consulting with patients (focus groups or in-depth interviews) and a literature review
Item selection	Determining the items included in the final instrument	**√√** A pilot instrument was developed and tested with Rasch or factor analysis and statistical justification provided for removing items, plus items with floor and ceiling effects removed and the amount of missing data considered
Scoring	A description of how the instrument should be scored	**√√** Rasch scoring of a statistically justified response scale
Views of stroke patients considered	The percentage of stroke patients involved in item identification during the development of PROMs	**√** Less than 50% of stroke patients were involved in the consultation with patients in the item identification
Stroke population	The extent to which the instrument has been studied in a stroke population	**√√** Stroke population studied

**√√** positive rating; **√** minimal acceptable rating; **X** negative rating

Three alternative instruments which had not previously been validated with stroke survivors were identified in this review; the Impact of Vision Impairment (IVI), the Vision-related Quality of Life (VQoL) questionnaire and Visual Symptom and Quality of Life (VSQ) questionnaire [54–56]. Both the IVI and VQoL measure frequency, i.e. “In the past month, how much has your eyesight interfered with …” [55, 56]. These questionnaires would not be appropriate for use soon after onset of the condition. An element of memory is also required to answer these items, which may cause difficulties in the presence of cognitive impairment. The VSQ does contain some items measuring frequency but this is not standardised throughout the whole instrument. During the development of the BIVI-IQ these elements were avoided, with all questions specifying “difficulty due to your eyes or eyesight” and the rating scale measuring the amount of difficulty experienced at the time of completion.

This study has been able to demonstrate the BIVI-IQ fulfils some of the criteria considered to be important when selecting a PROM [57, 58]. Unidimensionality has been demonstrated using Rasch analysis indicating internal consistency [57]. Face and content validity can be argued due to the employment of stroke survivors and clinicians at every stage of development [59]. This involvement also aids with creating an acceptable instrument. However, the BIVI-IQ-15 has not yet been assessed in its current form and the Rasch analysis presented in this study does not reflect the final version; appropriate psychometric validation of this should be included in a future validation study. Other criteria are yet to be satisfied and require testing in a further validation study; specifically precision, reproducibility, construct validity and responsiveness [57].

The BIVI-IQ was developed to be suitable for any of the four main categories of visual impairment. The population sample completing the version two pilot had a range of dependency as measured by the Barthel Index, indicating it is suitable to measure vision-related quality of life in the presence of other impairments. The BIVI-IQ would be suitable for use in routine practice or research settings. It is short, only taking a few minutes to fill out and easy to use for self-completion.

In cases of asymptomatic mild visual impairment (e.g. partial superior homonymous quadrantanopia) or a visual impairment for which the symptoms had been eliminated by treatment (e.g. diplopia joined with a prism), the visual impairment may not have any impact on the individuals’ vision-related quality of life. Further in-depth investigation is required for these groups to establish if an asymptomatic visual impairment does or does not affect individuals’ vision-related quality of life.

## Conclusion

Prior to this body of work, there were no PROMs specifically designed to measure the impact of visual impairment following stroke on quality of life. In conclusion, the BIVI-IQ questionnaire is presented; a 15-item instrument reporting quality of life for individuals with visual impairment related to stroke. An independent validation is now required to confirm that the BIVI-IQ is an effective tool across acquired brain injuries causing visual impairment. Future work will involve maximising interpretability and exploration of the barriers and solutions to implementation.

## Additional files

Additional file 1: Summary fit statistics for development process using Rasch analysis (DOCX 28 kb)

Additional file 2: Order of item deletion and data influencing decisions for deletion (DOCX 28 kb)

## Abbreviations

BIVI-IQ-15: Brain Injury related Visual Impairment Impact Questionnaire
DIF: Differential item functioning
IVI: Impact of Vision Impairment questionnaire
NEI VFQ-25: National Eye Institute Visual Function Questionnaire-25
PROM: Patient reported outcome measure
PSI: Person separation index
VQoL: Vision-related Quality of Life questionnaire
VSQ: Visual Symptom and Quality of Life questionnaire
