# Supplementary material for: Development of a patient reported outcome measures for measuring the impact of visual impairment following stroke
Source: BMC Health Serv Res. 2019 May 31;19:348. doi: 10.1186/s12913-019-4157-3 (PMC6544926; doi:10.1186/s12913-019-4157-3)
Supplement: Supplementary file 1 — Summary fit statistics for development process using Rasch analysis (DOCX 28 kb) [file 12913_2019_4157_MOESM1_ESM.docx]

**Additional File 1:** Summary fit statistics for development process using Rasch analysis

| **Analysis** | **Number of items** | **Item Fit Residual** | | **Person Fit Residual** | | **Item-trait Interaction** | | **Unidimensionality** | **PSI (**with extremes) |
| --- | --- | --- | --- | --- | --- | --- | --- | --- | --- |
|  |  | Mean | SD | Mean | SD | Chi square (df) | p | Percent <5% (95% CI) |  |
| Initial | 62 | 1.044 | 8.283 | 0.506 | 3.456 | 2110.3 (186) | <0.0001 | 55.9% | 0.955 |
| Rescore | 62 | -0.306 | 2.407 | -0.216 | 1.681 | 753.0 (186) | <0.0001 | 36.0% | 0.950 |
| Deletion: | | | | | | | | | |
| ‘Overall health’ | 61 | -0.227 | 2.002 | -0.267 | 1.743 | 483.0 (183) | <0.0001 | 28.7% | 0.950 |
| ‘Overall vision’ | 60 | -0.196 | 1.734 | -0.276 | 1.774 | 401.1 (180) | <0.0001 | 25.9% | 0.945 |
| ‘Dry eyes’ | 59 | -0.209 | 1.710 | -0.294 | 1.788 | 382.1 (177) | <0.0001 | 24.7% | 0.944 |
| ‘Watery eyes’ | 58 | -0.212 | 1.648 | -0.303 | 1.794 | 341.0 (174) | <0.0001 | 25.9% | 0.944 |
| ‘Double vision | 57 | -0.204 | 1.580 | -0.314 | 1.779 | 295.3 (171) | <0.0001 | 24.7% | 0.944 |
| ‘Deterioration of vision’ | 56 | -0.221 | 1.532 | -0.320 | 1.784 | 291.1 (168) | <0.0001 | 25.1% | 0.943 |
| ‘Eyes see differently’ | 55 | -0.224 | 1.485 | -0.327 | 1.790 | 264.4 (165) | <0.0001 | 22.7% | 0.942 |
| ‘Crossing the road’ | 54 | -0.214 | 1.420 | -0.325 | 1.763 | 256.0 (162) | <0.0001 | 24.3% | 0.941 |
| ‘Moving around in unfamiliar areas’ | 53 | -0.216 | 1.362 | -0.326 | 1.738 | 255.6 (159) | <0.0001 | 23.1% | 0.939 |
| ‘Limit on how long activities can be done for’ | 52 | -0.222 | 1.305 | -0.332 | 1.720 | 245.9 (156) | <0.0001 | 21.9% | 0.937 |
| ‘Loss of confidence’ | 51 | -0.226 | 1.237 | -0.339 | 1.707 | 232.9 (153) | <0.0001 | 20.2% | 0.935 |
| ‘Participating in indoor social activities’ | 50 | -0.221 | 1.187 | -0.336 | 1.686 | 212.9 (150) | <0.0001 | 20.2% | 0.933 |
| ‘Household chores’ | 49 | -0.213 | 1.137 | -0.330 | 1.660 | 194.4 (147) | 0.0005 | 21.9% | 0.932 |
| ‘Dealing with strangers’ | 48 | -0.201 | 1.095 | -0.325 | 1.654 | 180.4 (144) | 0.0053 | 20.7% | 0.931 |
| ‘Noticing objects off to one side’ | 47 | -0.171 | 1.107 | -0.311 | 1.630 | 232.3 (188) | 0.0215 | 19.4% | 0.927 |
| ‘Missing patches of vision’ | 46 | -0.180 | 1.096 | -0.309 | 1.610 | 183.6 (138) | 0.0155 | 17.8% | 0.925 |

| **Analysis** |  | **Item Fit Residual** | | **Person Fit Residual** | | **Item-trait Interaction** | | **Unidimensionality** | **PSI (**with extremes) |
| --- | --- | --- | --- | --- | --- | --- | --- | --- | --- |
|  |  | Mean | SD | Mean | SD | Chi square (df) | p | Percent <5% (95% CI) |  |
| ‘Bumps into or against objects or people in crowded areas’ | 45 | -0.185 | 1.075 | -0.310 | 1.592 | 159.3 (135) | 0.0058 | 21.1% | 0.923 |
| ‘Moving around outdoors’ | 44 | -0.191 | 1.033 | -0.311 | 1.568 | 152.9 (132) | 0.0753 | 19.8% | 0.921 |
| ‘Using a computer’ | 43 | -0.197 | 1.050 | -0.313 | 1.543 | 136.9 (129) | 0.1029 | 19.8% | 0.919 |
| 'Objects suddenly appearing’ | 42 | -0.205 | 1.055 | -0.314 | 1.529 | 145.3 (126) | 0.3001 | 20.7% | 0.917 |
| ‘Moving around on uneven ground’ | 41 | -0.193 | 1.054 | -0.317 | 1.522 | 134.0 (123) | 0.1153 | 17.8% | 0.914 |
| ‘Seeing in bright light’ | 40 | -0.214 | 1.063 | -0.315 | 1.488 | 130.8 (120) | 0.2342 | 17.4% | 0.912 |
| ‘Seeing the far side of a room’ | 39 | -0.221 | 1.065 | -0.318 | 1.472 | 133.5 (117) | 0.2349 | 15.4% | 0.910 |
| ‘Usual standard’ | 38 | -0.256 | 0.998 | -0.329 | 1.470 | 139.9 (114) | 0.1407 | 12.6% | 0.904 |
| ‘Bathing or showering’ | 37 | -0.241 | 0.970 | -0.327 | 1.469 | 130.8 (111) | 0.0512 | 13.8% | 0.903 |
| ‘Seeing in poor or dim light’ | 36 | -0.254 | 0.966 | -0.330 | 1.457 | 127.1 (108) | 0.1009 | 11.7% | 0.900 |
| ‘Toileting’ | 35 | -0.239 | 0.962 | -0.322 | 1.452 | 124.8 (105) | 0.0910 | 12.2% | 0.899 |
| ‘Eating’ | 34 | -0.228 | 0.963 | -0.312 | 1.464 | 119.7 (102) | 0.1116 | 13.8% | 0.898 |
| ‘Reading the same size print’ | 33 | -0.253 | 1.014 | -0.315 | 1.436 | 125.0 (99) | 0.0397 | 13.0% | 0.894 |
| ‘Stay at home’ | 32 | -0.249 | 0.992 | -0.318 | 1.430 | 118.2 (96) | 0.0620 | 11.3% | 0.892 |
| ‘Fluctuation’ | 31 | -0.261 | 1.014 | -0.313 | 1.401 | 116.4 (93) | 0.0505 | 11.3% | 0.890 |
| ‘Trips and falls’ | 30 | -0.25 | 0.964 | -0.305 | 1.371 | 114.2 (90) | 0.0435 | 11.3% | 0.887 |
| ‘Vulnerable’ | 29 | -0.243 | 0.988 | -0.300 | 1.338 | 121.7 (87) | 0.0083 | 10.5% | 0.881 |
| ‘Taking medication’ | 28 | -0.25 | 0.973 | -0.298 | 1.324 | 108.9 (84) | 0.0354 | 10.9% | 0.878 |
| ‘Pouring a drink’ | 27 | -0.242 | 0.998 | -0.297 | 1.328 | 108.7 (81) | 0.0219 | 9.3% | 0.875 |
| **Analysis** |  | **Item Fit Residual** | | **Person Fit Residual** | | **Item-trait Interaction** | | **Unidimensionality** | **PSI (**with extremes) |
|  |  | Mean | SD | Mean | SD | Chi square (df) | p | Percent <5% (95% CI) |  |
| ‘Writing’ | 26 | -0.263 | 1.027 | -0.301 | 1.307 | 122.1 (78) | 0.0011 | 9.3% | 0.870 |
| ‘Making eye contact’ | 25 | -0.246 | 0.969 | -0.294 | 1.293 | 111.1 (75) | 0.0043 | 8.9% | 0.866 |
| ‘Close-up vision’ | 24 | -0.274 | 1.028 | -0.299 | 1.280 | 106.8 (72) | 0.0049 | 8.1% | 0.861 |
| ‘Seeing faces’ | 23 | -0.264 | 1.059 | -0.291 | 1.254 | 96.8 (69) | 0.0153 | 6.9% (4.2-9.6%) | 0.858 |
| ‘Blurred vision’ | 22 | -0.267 | 1.035 | -0.298 | 1.238 | 81.5 (66) | 0.0941 | 6.9% (4.2-9.6%) | 0.852 |
| ‘Change in colour perception' | 21 | -0.266 | 1.087 | -0.297 | 1.239 | 84.3 (63) | 0.0379 | 4.1% | 0.851 |
| ‘Taking medication’ | 20 | -0.222 | 1.091 | -0.281 | 1.246 | 80.5 (60) | 0.0398 | 5.3% (2.5-8.0%) | 0.849 |
| ‘Not coping’ | 19 | -0.193 | 0.966 | -0.275 | 1.235 | 78.1 (57) | 0.0332 | 3.2% | 0.841 |

SD = standard deviation, df = degrees of freedom, 95% CI = 95% confidence intervals, PSI = person separation index.

For the data to indicate fit to the Rasch model:

- Perfect fit mean = 1 and SD = 0 - Tolerance range for fit residual mean +2.5 to -2.5 - Chi square value should be low and non-significant

|  | Misfit |  | Fit with adjustment |  | Fit without adjustment |
| --- | --- | --- | --- | --- | --- |
|  |  |  | (Bonferoni/95% CI) |  |  |

- For indication of unidinmensionality less than 5% of t-tests should be significant (0.05 with Bonferoni correction)

or the lower 95% CI should be less than 5%.

- PSI should be ≥0.7 to enable detection of at least two groups.
