# Supplementary material for: Development of a patient reported outcome measures for measuring the impact of visual impairment following stroke
Source: BMC Health Serv Res. 2019 May 31;19:348. doi: 10.1186/s12913-019-4157-3 (PMC6544926; doi:10.1186/s12913-019-4157-3)
Supplement: Supplementary file 2 — Order of item deletion and data influencing decisions for deletion (DOCX 28 kb) [file 12913_2019_4157_MOESM2_ESM.docx]

**Additional File 2:** Order of item deletion and data influencing decisions for deletion

| **Item deleted** | **Misfit** | **Differential Item Functioning (DIF)** | **Local dependence**  **(residual correlations)** | **Clinical** |
| --- | --- | --- | --- | --- |
| ‘Overall health’ | Fit residual = 12.78  Chi square *p*<0.0001  F-statistic *p*<0.0001 | Uniform  Time since stroke *p*<0.0001  Location *p*<0.0001 | ‘Overall vision’ 0.513 | Contribution to multidimensionality as not related to impact of visual impairment |
| ‘Overall vision’ | Fit residual = 8.984  Chi square *p*<0.0001  F-statistic *p*<0.0001 | Uniform  Time since stroke *p*<0.0001  Location *p*<0.0001 | ‘Deterioration of vision’ 0.239 | - |
| ‘Dry eyes’ | Fit residual = 3.949  Chi square *p*<0.0001 | - | - | - |
| ‘Watery eyes’ | Fit residual = 4.100  Chi square *p*<0.0001  F-statistic *p*<0.0001 | - | - | - |
| ‘Double vision’ | Fit residual = 3.882  Chi square *p*<0.0001  F-statistic *p*=0.0002 | Uniform  Primary visual impairment *p*<0.0001 | ‘Judging distances’ 0.214 | - |
| ‘Deterioration of vision’ | Fit residual = 3.707 | - | ‘Blurred vision’ 0.321  ‘Fluctuation’ 0.212  ‘Seeing far side of room’ 0.212 | No specifics regarding deterioration over what period of time and if this is pre or post-stroke onset |
| ‘Eyes seeing differently’ | Fit residual = 3.476  Chi square *p*<0.0001 | - | ‘Fluctuation’ 0.257  ‘Judging distances’ 0.186 | Does not specify if it asking regarding each eye separately at the same time point or both eyes together over different time points |

| **Item deleted** | **Misfit** | **Differential Item Functioning (DIF)** | **Local dependence**  **(residual correlations)** | **Clinical** |
| --- | --- | --- | --- | --- |
| ‘Crossing the road’ | Fit residual = 3.151 | Uniform  Location *p*<0.0001 | ‘Moving around on uneven ground’ 0.243  ‘Moving around in unfamiliar places’ 0.355  ‘Moving around outdoors’ 0.380 | - |
| ‘Moving around in unfamiliar areas’ | Fit residual = -2.919 | Uniform  Location *p*<0.0001 | ‘Moving around on uneven ground’ 0.264  ‘Trips and falls’ 0.210  ‘Moving around in familiar areas’ 0.258  ‘Bumps into or against objects or people in crowded areas’ 0.384  ‘Moving around outdoors’ 0.265 | - |
| ‘Limit of how long activities can be done for’ | Fit residual = -2.961 | - | ‘Loss of confidence’ 0.240  ‘Accomplishing as much as would like’ 0.436  ‘Usual standard’ 0.322 | - |
| ‘Loss of confidence’ | Fit residual = -2.725 | - | ‘Accomplishing as much as would like’ 0.438  ‘Usual standard’ 0.269 | - |
| ‘Participating in indoor social activities’ | Fit residual = -2.520  F-statistic *p*=0.0003 | - | ‘Making eye contact’ 0.218  ‘Dealing with strangers’ 0.223  ‘Participating in outdoor social activities’ 0.447 | - |
| ‘Household chores’ | Chi square *p*=0.0007  F-statistic *p*<0.0001 | - | ‘Preparing something to eat’ 0.251  ‘Looking after appearance’ 0.207 (53) ‘Shopping’ 0.520 | - |
| ‘Dealing with strangers’ | F-statistic *p*=0.0006 | - | ‘Making eye contact’ 0.401  ‘Participating in outdoor social activities’ 0.187 | ‘Making eye contact’ would cover people the person knows as well as strangers |
| **Item deleted** | **Misfit** | **Differential Item Functioning (DIF)** | **Local dependence**  **(residual correlations)** | **Clinical** |
| ‘Noticing objects off to the side’ | - | Uniform  Primary visual impairment *p*<0.0001  Location *p*<0.0001 | ‘Objects suddenly appearing’ 0.349  ‘Missing patches of vision’ 0.571 (33) ‘Bumps into or against objects or people in crowded areas’ 0.268 | - |
| ‘Missing patches of vision’ | - | Uniform  Primary visual impairment *p*<0.0001 | ‘Objects suddenly appearing’ 0.486  ‘Bumps into or against objects or people in crowded areas’ 0.306 | - |
| ‘Bumps into or against objects or people in crowded areas’ | - | Uniform  Location *p*<0.0001 | ‘Objects suddenly appearing’ 0.239  ‘Moving around on uneven ground’ 0.296  ‘Trips and falls’ 0.219  ‘Moving around outdoors’ 0.218 | - |
| ‘Moving around outdoors’ | - | Uniform  Location *p*<0.0001 | ‘Moving around on uneven ground’ 0.207  ‘Participating in outdoor social activities’ 0.219  ‘Shopping’ 0.226  ‘Stay at home’ (0.183) | - |
| ‘Using a computer’ | - | Non-uniform  Time since stroke *p*<0.0001 | ‘Following a line of print’ 0.188 | ‘Using a computer’ involves reading. ‘Following a line of print’ also covers printed mediums |
| ‘Objects suddenly appearing’ | - | Uniform  Primary visual impairment *p*<0.0001 | - | - |
| ‘Moving around on uneven ground’ | - | Uniform  Location *p*<0.0001 | ‘Trips and falls’ 0.239 | - |
| ‘Seeing in bright light’ | - | Uniform  Location *p*=0.0003 | ‘Seeing in poor or dim light’ 0.302 ‘Adjusting to differing lighting’ 0.586 | ‘Adjusting to differing lighting’ covers both bright and dim lighting conditions |

| **Item deleted** | **Misfit** | **Differential Item Functioning (DIF)** | **Local dependence**  **(residual correlations)** | **Clinical** |
| --- | --- | --- | --- | --- |
| ‘Seeing far side of room’ | - | - | ‘Seeing something far away’ 0.508  ‘Seeing faces’ 0.253 | ‘Seeing something far away’ more appropriate to cover the difficulty with reduced vision in the distance |
| ‘Usual standard’ | - | - | ‘Accomplish as much as would like’ 0.493 | ‘Accomplishing as much as would like’ more appropriate to cover the difficulty with achieving tasks a person needs to achieve |
| ‘Bathing or showering’ | - | - | ‘Moving around in familiar areas’ 0.296  ‘Toileting’ 0.492  ‘Getting dressed’ 0.437  ‘Preparing something to eat’ 0.320 | Of the three ADLs involved with personal hygiene, ‘getting dressed’ potentially most dependent on vision |
| ‘Seeing in poor or dim light’ | - | - | ‘Adjusting to differing lighting 0.435 | ‘Adjusting to differing lighting’ covers both bright and dim lighting conditions |
| ‘Toileting’ | - | - | ‘Getting dressed’ 0.496  ‘Preparing something to eat’ 0.229 | ‘Getting dressed’ potentially most dependent on vision |
| ‘Eating’ | - | - | ‘Pouring a drink’ 0.423 | - |
| ‘Reading same print size’ | - | - | ‘Following a line of print’ 0.378 | ‘Following a line of print’ more commonly occurs following stroke in both ocular motility defects and visual field loss. Element of ‘reading same size print’ which relies on memory |
| ‘Stay at home’ | - | Non-uniform  Time since stroke *p*=0.0003 | ‘Participating in outdoor social activities’ 0.224  ‘Shopping’ 0.251  ‘Not coping’ 0.174 | - |

| **Item deleted** | **Misfit** | **Differential Item Functioning (DIF)** | **Local dependence**  **(residual correlations)** | **Clinical** |
| --- | --- | --- | --- | --- |
| ‘Fluctuation’ | - | - | ‘Blurred vision’ 0.331  ‘Objects jumping around’ 0.190 | No specifics of fluctuation over what period of time and if this is pre- or post-stroke onset |
| ‘Moving around in familiar areas’ | - | - | ‘Moving around indoors’ 0.329 | - |
| ‘Vulnerable’ | - | - | ‘Negative emotions’ 0.279  ‘Burden to others’ 0.172 | ‘Negative emotions’ a more general item about emotional well-being |
| ‘Preparing something to eat’ | - | - | ‘Getting dressed’ 0.245  ‘Taking medication’ 0.231  ‘Pouring a drink’ 0.196  ‘Looking after appearance’ 0.197  ‘Shopping’ 0.184 | ‘Preparing something to eat’ is commonly not possible in an inpatient setting |
| ‘Pouring a drink’ | - | - | ‘Taking medication’ 0.171  ‘Looking after appearance’ 0.241 | - |
| ‘Writing’ | - | - | ‘Close up vision’ 0.222  ‘Following a line of print’ 0.238 | More appropriate to have an item relating to reading ‘following a line of print’ rather than ‘writing’ |
| ‘Making eye contact’ | F-statistic *p*=0.0017 | - | - | - |
| ‘Close up vision’ | - | - | ‘Seeing faces’ 0.223  ‘Following a line of print’ 0.231 | More appropriate to retain an item related to reading ‘following a line of print’ |
| ‘Seeing faces’ | - | - | ‘Seeing something far away’ 0.235 | ‘Seeing something far away’ more general item, more appropriate to assess the impact of reduced vision |
| ‘Blurred vision’ | - | - | ‘Seeing something far away’ 0.220 | More appropriate ‘seeing something far away’ to cover the difficulties with reduced vision |
| **Item deleted** | **Misfit** | **Differential Item Functioning (DIF)** | **Local dependence**  **(residual correlations)** | **Clinical** |
| ‘Change in colour perception’ | - | - | ‘Adjusting to differing lighting’ 0.213 | More stroke survivors complain of difficulty with lighting than colour |
| ‘Taking medication’ | - | - | ‘Looking after appearance’ 0.190 | - |
| ‘Not coping’ | - | - | ‘Self-conscious’ 0.154 | - |
